# Expression of Ubiquitin-specific protease 7 in oral squamous cell carcinoma promotes tumor cell proliferation and invasion

**DOI:** 10.1590/1678-4685-GMB-2021-0058

**Published:** 2021-11-22

**Authors:** Xiaojie Yang, Jiamin Jin, Jinfeng Yang, Lihua Zhou, Sisi Mi, Guangying Qi

**Affiliations:** 1 Guilin Medical University, Laboratory of Tumor Immunology and Microenvironmental Regulation, Guilin, Guangxi, China.; 2 Hospital of Guangxi Medical University, Department of Pathology, Nanning, Guangxi, China.; 3 Guilin Medical University, Department of Immunology, Guilin, Guangxi, China.

**Keywords:** Oral Squamous Cell Carcinoma, USP7, siRNA, proliferation, invasion

## Abstract

Oral Squamous Cell Carcinoma (OSCC) is the most common malignant cancer affecting oral cavity. Recent studies have demonstrated that Ubiquitin-specific protease 7 (USP7) was upregulated in several types of cancers. USP7 expression was associated with various proto-oncogenes and tumor suppressor genes. However, USP7 expression level and its functional role in OSCC is unclear. In the current study, we showed that USP7 expression in OSCC tissues was generally upregulated compared to normal adjacent tissues by using IHC. Furthermore, statistical analysis uncovered that USP7 expression was positively correlated with Ki-67, MMP2, VEGF in OSCC tissues. Importantly, high USP7 expression was significantly correlated with lymph node metastasis and histological differentiation in OSCC patients. So, our hypothesis is that USP7 plays a tumor-promoting role in OSCC. Knocking down of USP7 in tumor cells not only suppressed HSC3 cells proliferation, migration and invasion, but also promoted cell apoptosis. Moreover, USP7 siRNA blocked the activation of Akt/ERK signaling pathway. In conclusion, data presented here suggests that USP7 promotes the progression of OSCC. USP7 may be used as a new therapeutic target for OSCC diagnosis and treatment.

## Introduction

Oral cancer is a type of severe malignant head and neck tumor, with about 90% oral cancer cases are Oral Squamous Cell Carcinoma (OSCC) (Bray et al., 2018). There were about 70 million patients diagnosed with OSCC worldwide in the past five years (Jason et al., 2020). The 5-year survival rate is about 39% (Zhang et al., 2019). The prognosis of OSCC is still poor because of the lack of effective therapeutic target genes (Haddad and Shin, 2008; Wikner et al., 2014). Thus, there is urgent need to investigate the pathogenesis and molecular mechanism of OSCC occurrence and progression for further improvement of the survival and life quality.

The ubiquitin proteasome system is important for maintaining protein turnover for cancer progression (Young et al., 2019). We have previously demonstrated that USP22 was upregulated in OSCC and hepatocellular carcinoma (Tang et al., 2015; Liu et al., 2019). USP22 expression is a positively correlated with Aurora-B and Survivin expression. USP22 can also regulate the expression of cell cycle related proteins in order to promote cancer cell growth (Liu *et al.*, 2019). Recent studies show that USP7, which is involved in tumor-genesis and process (Song et al., 2008; Zhang et al., 2016), could be used for novel drug target in cancer therapy (Zhou et al., 2018). USP7 expression is not only closely related to tumor cell proliferation and invasion (Chiu et al., 2014), but also affects some critical signaling pathways (Zhou *et al.*, 2018). However, the underlying mechanism of how USP7 affects these processes in OSCC remains unclear.

USP7, an important member in ubiquitin-specific protease family, was first discovered in 1997 (Everett et al., 1997). USP7 can regulate the expression of various tumor-related genes, such as p53 and Ki-67 (Sarkari et al., 2010; Zhou et al., 2018). USP7 promotes the proliferation of non-small cell lung cancer cell via stabilizing Ki-67 protein (Zhang et al., 2016). Knockdown of USP7 expression increases p53 expression and inhibits colon cancer cells proliferation (Colland et al., 2009). OSCC patients with high Ki-67 expression showed a significantly increased risk of poor overall survival as well as disease-free survival (Jing et al., 2018). In addition, invasion-metastasis-related factor matrix metalloproteinase 2 (MMP2) was shown to improve OSCC migration and invasion (Celentano et al., 2021); Angiogenesis was also involved in OSCC progression. Vascular endothelial growth factor (VEGF) and its receptor VEGFR are the main factors that are responsible for angiogenesis (Mărgăritescu et al., 2009). VEGF is highly expressed and considered as a marker of OSCC progression (Mărgăritescu *et al.*, 2010). However, the relationship of USP7 with Ki-67, MMP2, and VEGF in OSCC is still unclear.

Therefore, aim of the current study is to investigate the molecular mechanism of USP7 in regulating tumor progression and signaling pathways of OSCC and to verify its relationship with Ki-67, MMP2, and VEGF.

## Material and Methods

### Patient samples

92 cases of surgical OSCC specimens were collected and sorted from the Department of Pathology in the Affiliated Hospital for Guilin Medical University (Guilin, Guangxi, China) from 2010 to 2017. All steps conducted were following the ethical standards of the institutional and national research committee, the 1964 Declaration of Helsinki, and its later amendments or comparable ethical standards. The research was approved by Guilin Medical University (Guilin, China) Ethics Committee. Written informed consent was provided by every participant involved in this work. The complete clinical and pathological characteristics were recorded, including age, gender, tissue histological differentiation and lymph node metastasis. Before operation, the patient were confirmed with no radiotherapy or chemotherapy treatment, and all had a definite pathological diagnosis of OSCC after operation. The normal control tissues, which were more than 5 cm away from the tumor site of the same patients were collected from oral mucosal tissues by using naked eye.

Immunohistochemical staining

All specimens were first immersed and fixed in neutral formalin. Later tissues were embedded with paraffin, then made into 4 μm thick continuous sections. The sections were routinely dew-axed and hydrated. The EDTA antigen repair solution was boiled for 2 minutes, then was allowed to cool to room temperature. The tissue sections were blocked with an endogenous peroxidase blocker at 37 °C for 15 min and incubated with polyclonal anti-USP7 antibody (rabbit, cat.# ab4080; 1:1000; Abcam, USA), monoclonal anti-Ki-67 antibody (mouse, cat.# ZM-0166; 1:800, zsbio, China), monoclonal anti-MMP2 antibody (mouse, cat.# ab86607; 1:1000; Abcam, USA), and monoclonal anti-VEGF antibody (rabbit, cat.# ab155288; 1:1000; Abcam, USA) overnight at 4 °C respectively. HRP-conjugated anti-rabbit IgG or AP-conjugated anti-mouse IgG (cat.# DS-0004; zsbio, China) antibodies was incubated at 37 °C for 30 min. DAB substrate was added and the obtained result was observed under a microscope (x 200 magnification; light microscope; Olympus BX53F). USP7, Ki-67, MMP2 and VEGF expression were graded high (++~+++) or low (-~+) based on the proportion of positive tumor cells (positive cells: >50% are strongly positive (+++); 20-50% are moderately positive (++); 5-20% are weakly positive (+) ; <5% are negative (-). 

### Cell culture and treatment

Human OSCC cell lines (HSC3, HSC4, KB) were all purchased from the American Tissue Culture and Preservation Center (ATCC). HSC3 and HSC4 cells were cultured with RPMI-1640 complete medium that have 10% fetal bovine serum (FBS) and 1% penicillin streptomycin (PS). KB cells were cultured with DMEM (10% FBS, 1% PS). The medium’s placement condition was 37 °C, 5% CO_2_ for cultivation.

### Western Blot

Cells were lysed by RIPA buffer for 30 min together with protease inhibitor, the samples were centrifuged at 11000 g for at 4 °C 15 min. Twenty microgram of protein was loaded to SDS-PAGE and transferred to polyvinyl difluoride (PVDF) membrane (Bio-Rad Laboratories, USA). The membrane was subjected to primary antibodies (4 ± 0.5 °C, overnight) and secondary antibodies (1 h at room temperature) respectively. Primary antibodies contained USP7 (rabbit; cat.# ab4080; 1:1000; Abcam; USA), Cyclin A (mouse; cat.# SC-271682; 1:500; Santa Cruz Biotechnology; USA) , cleaved Caspase 3 (rabbit; cat.# 9661; 1:1000; Cell Signaling Technology; USA), Caspase 3 (rabbit; cat.# 19677-1; 1:1000; Proteintech Group (PG); China), Bax (rabbit; cat.# 60267-1-Ig; 1:1000; PG; China), Bcl-2 (rabbit; cat.# 60178-1-Ig; 1:1000; PG; China), AKT(mouse; 60203-2-Ig; 1:1000; PG; China), p-AKT(mouse; 66444-1-Ig; 1:1000; PG; China), ERK(Rabbit;cat.#11257-1-AP;1:1000;PG;China), p-ERK(Rabbit;cat.#28773-1-AP; 1:5000;PG;China). The secondary antibodies contained goat anti-mouse HRP (cat.# ZB-2305; 1:10000, zsbio, China) or goat anti-rabbit HRP (cat.# ZB-2301; 1:10000, zsbio, China). GAPDH (mouse; cat.# TA-08; 1:1000; zsbio; China) was used as house-keeping gene control.

### Knockdown of USP7 with small interfering RNA (siRNA) transfection

Three USP7 siRNA were designed and transfected. The sequences were listed below: siRNA-USP7-Homo-603 (5’- GCAGUGCUGAAGAUAAUAATT- 3’, 5’ -UUAUUAUCUUCAGCACUGCTT-3’); siRNA-USP7-Homo-2033 (5’ - CAUGGGAUUUCCACAAGAUTT- 3’, 5’- AUCUUGUGGAAAUCCCAUGTT-3’ ); siRNA-USP7-Homo-3359 (5’ to 3’- GCCCGGUAAUAUGUCUCAUTT, 5’- AUGAGACAUAUUACCGGGCTT-3’ ); negative control (5’ -UUCUCCGAACGUGUCACGUTT-3’, 5’- ACGUGACACGTTCGGAGAA- 3’) (Gene Pharma, China). HSC3, HSC4, KB cells were transfected with siRNA-USP7 or negative control by using Lipofectamine^®^ 3000 Transfection reagent (Thermo Fisher Scientific, Inc.).

### CCK-8 assay

Cell suspensions of the blank group, negative control group and USP7 siRNA transfection group were inoculated in 96-well plates (each well contains about 1000 cells in 200 μl medium). Each group has 6 replicate wells. The cells were cultured in a carbon dioxide incubator at 37 °C. Twenty μl CCK-8 solution was added for 4h to each well at interval of 0h, 24h, 48h, and 72h after culture respectively. OD values were measured by absorbance at 450 nm with microplate.

### Wound-healing assay

Cell suspensions of the blank group, siRNA control group and USP7 siRNA transfection group were plated in 6-well plate (each well contains about 5×10^5^ cells) for 24 h. One hundred microliter sterile pipette tips were used to scribe a line evenly in the 6-well plate, and photographs were taken at 0 h and 24 h after scratching to observe the wound-healing ability of the relative cells.

### Transwell migration and invasion assay

Cell suspensions of the blank group, siRNA control group and USP7 siRNA transfection group were plated in Transwell chambers (24-well chambers, Corning Inc.). For migration assay, 100 µl cell (about 5×10^4^ cells/ml with serum-free medium) suspension was plated to the upper chamer, 600 µl of medium containing 20% FBS was added to the bottom chamber. The result was analyzed according to published literature (Zeng et al., 2019); For invasion assay, Matrigel (BD Biosciences) was first diluted at 1:9 ratio before it was added to the upper chamber. Cells and medium were performed the same as the migration assay. The migrated cells were stained with crystal violet 48 h later. Each sample was randomly counted in three fields (100x magnification) to evaluate the invasion ability of HSC3 cells. The result was analyzed according to published literature (Zeng *et al.*, 2019).

### Statistical analysis

All experiments were performed three times under the same conditions. The experimental data were analyzed by using SPSS v. 20.0 (SPSS, Inc, Chicago, USA) and GraphPad Prism 8. The data of multiple experiments were expressed in the form of mean ± standard deviation. The χ^2^-test was used for the comparison of numerical data. The data were analyzed by chi-square test and Spearman correlation analysis. The *t*-test compares the differences among the groups. *P<0.05* indicates that the difference between the groups was statistically significant.

## Results

### Expression of USP7, Ki-67, MMP2 and VEGF were elevated in OSCC tissues

**Figure 1 -:**
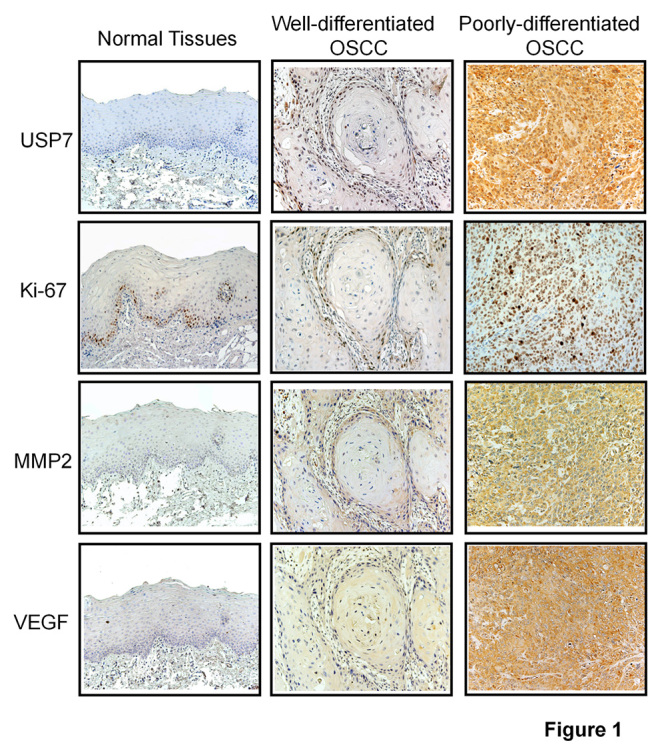
Expression of USP7, Ki-67, MMP2 and VEGF were elevated in OSCC tissues. Staining antibodies against human USP7, Ki-67, MMP2 and VEGF to show relative protein expression in normal tissues, well-differentiated OSCC and poorly-differentiated OSCC tissues. Image magnification is 200 times.

To clarify the role of USP7 in OSCC progression, the expression level of USP7, Ki-67, MMP2 and VEGF were examined in 92 OSCC tissues and 20 normal tissues by using immunohistochemistry staining. The expression of USP7, Ki-67, MMP2 and VEGF were mainly restricted to the basal layers of OSCC specimen (Figure 1). In contrast, there was weak expression of the above proteins in normal oral mucosa. Interestingly, the expression of these proteins was significantly elevated in poorly differentiated OSCC samples compared to well-differentiated samples (Figure 1). USP7 and Ki-67 were primarily localized in the nuclei, while MMP2 and VEGF were localized in the cytoplasm. These findings demonstrated that USP7 expression was elevated in OSCC tissues, suggesting that USP7 and its relative genes Ki-67, MMP2 and VEGF may promote the development of OCSS.

### Expression of USP7, Ki-67, MMP2 and VEGF were correlated with the clinicopathological features of OSCC

USP7 was highly expressed in 56 OSCC cases and poorly expressed in the other 36 OSCC cases, VEGF was highly expressed in 52 OSCC cases and poorly expressed in the other 40 OSCC cases (Table 1). The expression level of Ki-67 and MMP2 did not show significant differences in OSCC cases (Table 1). Among 56 cases with high USP7 expression, 39 cases showed high Ki-67, MMP2 and VEGF expression. Meanwhile, among 36 cases with low USP7 expression, 25 cases showed low Ki-67, MMP2 and VEGF expression (Table 2). These data indicated that USP7 expression was positively associated with Ki-67, MMP2 and VEGF expression levels.

**Table 1 -:** Differential expression analysis of USP7, Ki-67, MMP2 and VEGF in OSCC and normal tissues.

	Normal Tissues	OSCC	*P*-value
**USP7^a^ expression**			
Low	20	36	*<0.01*
High	0	56	
**Ki-67 expression**			
Low	20	45	
High	0	47	
**MMP2^b^ expression**			
Low	20	45	
High	0	47	
**VEGF^c^ expression**			
Low	20	40	*<0.01*
High	0	52	

^a^USP7, Ubiquitin-specific peptidase 7; ^b^MMP2, matrix metalloproteinase 2; ^c^VEGF, vascular endothelial growth factor

**Table 2 -:** Correlation of USP7 with Ki-67, MMP2 and VEGF expression in OSCC cases.

	USP7 expression		*P*-value
	Low 36	High 56	
**Ki-67 expression**			
Low	33	12	*<0.01*
High	3	44	
**MMP2 expression**			
Low	30	17	*<0.01*
High	6	39	
**VEGF expression**			
Low	25	17	*<0.01*
High	11	39	

Subsequently, USP7 expression was correlated to histological differentiation (*P*<0.01) and lymph node metastasis (*P*<0.05) (Table 3). High USP7 expression level was related to poorly differentiated OSCC tissues compared to well differentiated OSCC tissues (Table 3). These data suggested that USP7 expression was more prone to lymph node metastasis and poorly differentiated OSCC patients, indicating that USP7 overexpression was closely related to the malignancy of OSCC.

**Table 3 -:** USP7 expression and its correlation with clinicopathological features in OSCC cases.

	USP7 expression		*P*-value
	Low 36	High 56	
**Age (years)**			
≧50	27	43	
<50	9	13	
**Sex**			
Male	18	48	
Female	18	8	
**Lymph node metastasis**			
Negative	27	31	<0.05
Positive	9	25	
**Histological differentiation**			
Poor	0	24	<0.01
Well	36	32	

### USP7 knockdown suppresses OSCC cell proliferation and induces cell apoptosis

**Figure 2 -:**
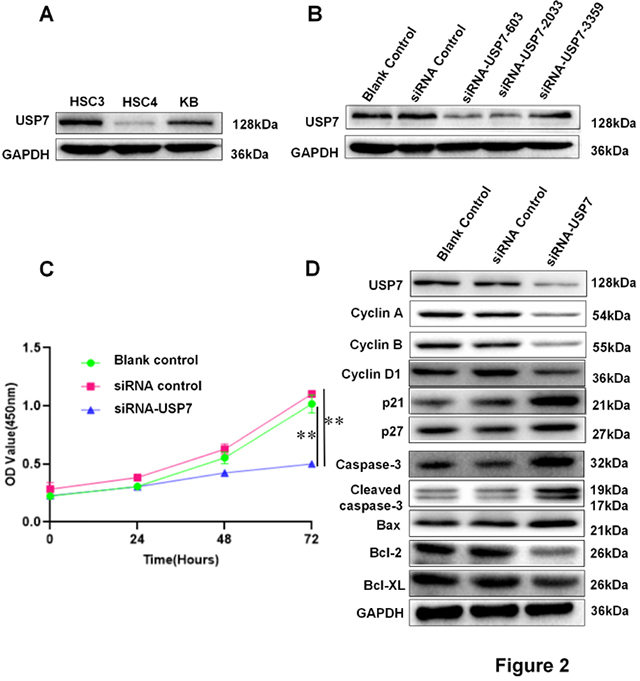
USP7 promotes OSCC cell proliferation by affecting cell cycle-related and apoptosis-related proteins. **A.** Western blot was used to detect the expression level of USP7 in HSC3, HSC-4, and KB cells. GAPDH was used as control. **B.** USP7 expression in HSC3 cell that was transfected with USP7 siRNA or siRNA negative control, and blank control. GAPDH was used as house-keeping control. **C.** Growth curve for HSC3 cells with siUSP7 treatment. Forty-eight hours after USP7 siRNA treatment, 1000 cells were seeded onto 96-well plate. Cell number at 24 h was set as 0 h. Cell numbers were continuously counted at 24 h, 48 h, 72 h. ** *P<0.01*. **D.** HSC3 cells were transfected with USP7 siRNA. Cell cycle-related and apoptosis-related genes were examined by WB at 48 h.

In order to explore the role of USP7 in OSCC, we firstly examined USP7 expression in HSC3, HSC4 and KB cell lines by using western blot. HSC3 cells had the highest expression of USP7 protein (>Figure 2A). So HSC3 cells were used for the following experiments. Three USP7 siRNA sequences (siRNA-USP7-603, siRNA-USP7-2033, siRNA-USP7-3359) were transfected respectively into HSC3 cells. USP7 expression was detected by western blot after transfection. Compared to the other two sequences, siRNA-USP7-603 (siUSP7) was shown to diminish USP7 expression most efficiently (Figure 2B). Thus siRNA-USP7-603 was used for the following experiments.

Next, we examined the influence of USP7 knockdown on the proliferation of OSCC cell by CCK-8 assay. The proliferation of HSC3 cells was significantly decreased in the USP7 siRNA group compared to blank control and siRNA control groups (Figure 2C). Cyclin A, Cyclin B and Cyclin D1 expression in HSC3 cells were reduced heavily in siUSP7 group. In contrast, the expression cyclin-dependent kinase inhibitors, p21 and p27 were increased in siUSP7 group (Figure 2D). The expression of pro-apoptotic factors Caspase-3, cleaved Caspase-3 and Bax were all increased, whereas the apoptosis inhibiting proteins Bcl-2 and Bcl-XL were decreased in the siUSP7 group (Figure 2D). These data suggested that USP7 knockdown inhibited HSC3 cell proliferation by regulating cell cycle and cyclin-dependent kinase inhibitors-related proteins, thus promoting HSC3 cells apoptosis by upregulating pro-apoptotic related protein.

### Suppression of OSCC cell migration and invasion by USP7 knockdown

**Figure 3 -:**
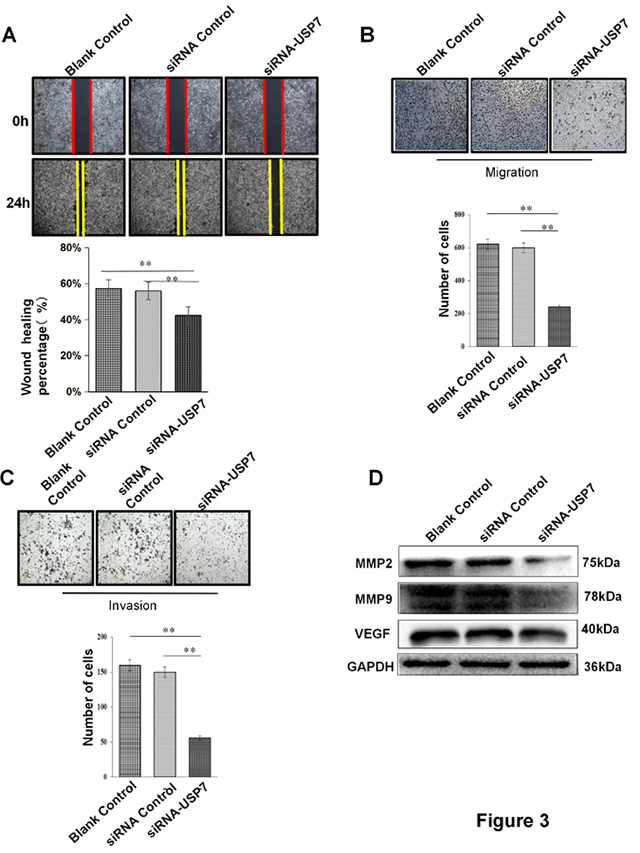
USP7 knockdown suppresses migration and invasion of OSCC cells *in vitro.* **A.** Wound healing assay showed migrating ability was inhibited by USP7 knockdown in HSC3 cell line. **B**. Tranwell assay demonstrated that USP7 knockdown inhibited the migration ability of HSC3 cells. Statistics on the number of cell metastases after USP7 knockdown in HSC3 cells. **C**. Transwell test was performed to detect HSC3 cell invasive ability after USP7 knockdown. **D**. The relative gene expression of MMP2, MMP9, VEGF in HSC3 cells transfected with USP7 siRNA or siRNA negative control, blank control, were determined by WB analysis. GAPDH was used as a control. Data were presented as mean ± SEM, **P<0.01.

The migration and invasion abilities were examined to investigate how USP7 may influence OSCC cells malignancy. Wound-healing assay was conducted to determine cell migration. The migration of HSC3 cells with siUSP7 was substantially reduced compared with blank and siRNA control groups (Figure 3A). Transwell assays showed that USP7 knockdown obviously suppressed OSCC cells migration and invasion compared to control groups (Figure 3B-C). The expression of migration and invasion related factors MMP2, MMP9 and VEGF were also decreased after USP7 knockdown in HSC3 cells (Figure 3D). These data were in line with previous results from immunohistochemistry staining in OSCC specimens, suggesting that the migration and invasion abilities were inhibited significantly after USP7 knockdown. 

### USP7 promotes malignancy of OSCC cells via activating Akt/ERK signaling pathway

**Figure 4 -:**
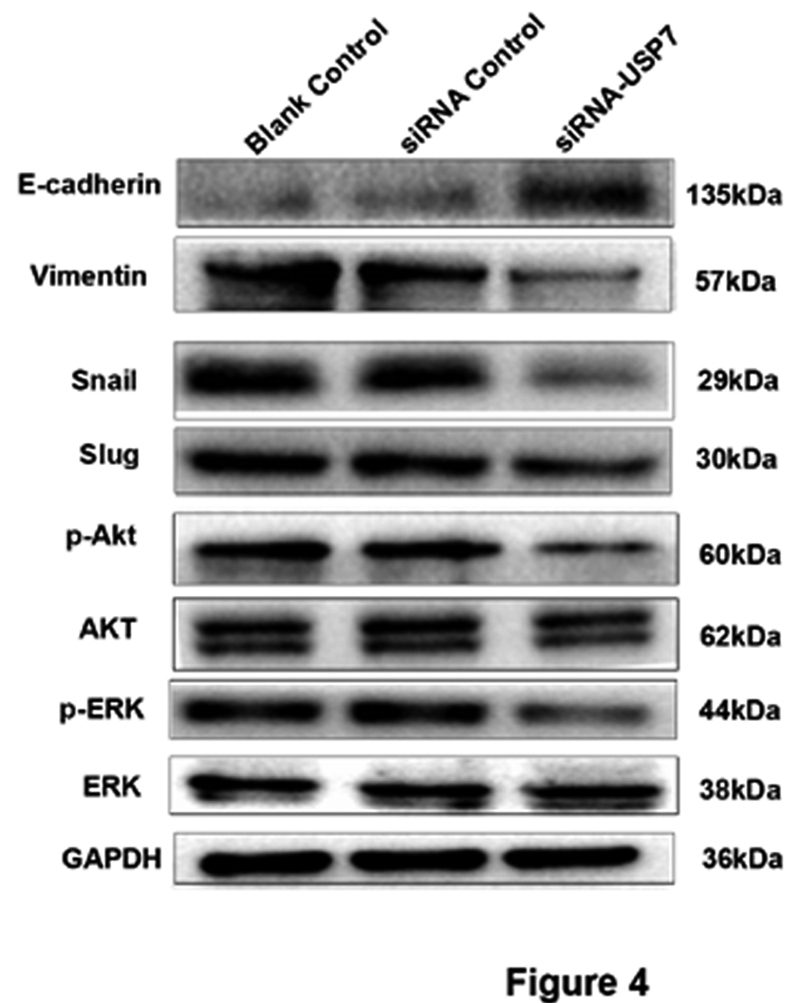
USP7 induces EMT of OSCC cells via driving the Akt/ERK signaling pathway. The expression of E-cadherin, vimentin, Snail, Slug, ERK and AKT pathway proteins in HSC3 cells with USP7 siRNA or siRNA negative control, blank control were determined by WB. GAPDH expression was used as a control.

The present data showed that USP7 could promote OSCC cell migration and invasion. But the underlying mechanism is not well defined. It is well known that epithelial-mesenchymal transition (EMT) is involved in OSCC cell invasion and metastasis (Cai et al., 2019). So the role of USP7 in EMT was examined. E-cadherin expression was upregulated and Vimentin, Snail, and Slug expression were all decreased in OSCC cells with USP7 knowdown (Figure 4). These data suggested that USP7 increased cell migration and invasion abilities by inducing EMT of OSCC cells.

Akt/ERK signaling pathway has been reported to be involved in OSCC proliferation, invasion and migration (Chiu et al., 2014; Chen et al., 2019). Here we investigated the effects of USP7 in the development of OSCC through Akt/ERK signaling pathway. In HSC3 cells with USP7 knockdown, p-Akt and p-ERK expression were all significantly decreased. These findings suggested that USP7 modulated malignancy of OSCC cells via activating Akt/ERK signaling pathway (Figure 4).

## Discussion

USP7 expression was correlated with poor outcome of several cancers, such as non small cell lung cancer, large cell carcinoma, and lung squamous cell carcinoma (Masuya et al., 2006; Morra et al., 2015). The current study demonstrated that the expression of USP7 was relatively low in normal oral mucosa tissues adjacent to cancer tissues, but showed a high expression level in OSCC tissues, especially in poorly differentiated carcinomas (Figure 1 and Table 1). Notably, the expression of USP7 is positively correlation with Ki-67, MMP2, VEGF expression in OSCC cases (Table 2), USP7 expression was closely related to the degree of OSCC lymph node metastasis and histological differentiation (Table 3). Therefore, USP7 may serve as a novel and potential therapeutic target in OSCC. 

The essential role of USP7 during cell cycle progression and apoptosis has been reported by other groups: i) USP7 knockdown leads to increased p53 expression and arrest of cell cycle (Cummins et al., 2004), ii) USP7 regulated various tumor-related factors expression (p53, Ki-67) in bladder cancer and prostate cancer (Song et al., 2008; Morra et al., 2019). So its expression is closely related to tumor cell proliferation. USP7 can stabilize Ki-67 protein through inhibiting its deubiquitination function (Zhang et al., 2016).

In the present study, the proliferation of HSC3 cells was inhibited in OSCC cells with USP7 knockdown (Figure 2C); Cyclin A, Cyclin B, and Cyclin D1 proteins were decreased, whereas p21 and p27, the cyclin-dependent kinase inhibitors were all increased (Figure 2D); Caspase-3, cleaved Caspase-3 and Bax proteins were increased and the expression of Bcl-2 and Bcl-XL, the apoptosis-inhibiting proteins were decreased (Figure 2D). These data indicate that USP7 was associated with the development of OSCC by inducing cell proliferation through activating cell cycle and inhibiting cell apoptosis.

On the other hand, it is widely known that USP7 overexpression in prostate cancer, ovarian cancer and liver cancer is highly associated with tumor metastasis and invasion (Nicholson and Suresh Kumar, 2011; Ma and Yu, 2016). Wound-healing assay and Transwell test have shown that OSCC cells transfected with USP7 siRNA could decrease migration and invasion abilities (Figure 3 A-C). WB results showed that USP7 was positively associated to MMP2, MMP9 and VEGF expression in HSC3 cells. In OSCC cells with USP7 knockdown, MMP2, MMP9 and VEGF proteins were also decreased (Figure 3D).

It is well-known that the activation of the Akt/ERK pathway is frequently detected in OSCC (Mishima et al., 2002). Whether USP7 can directly participate in the regulation of these pathways is currently unclear. WB results showed that USP7 silencing decreased the expression level of p-Akt, p-ERK, Vimentin, Snail, Slug proteins (Figure 4), indicating that USP7 may affect the Akt/ERK pathways, thereby affect the EMT of OSCC.

In conclusion, the current study indicates that USP7 is weakly expressed in normal oral mucosa tissues but with a high expression in OSCC tissues. Its expression is positively associated to migration and invasion-related factors MMP2, MMP9 and VEGF. USP7 is closely related to the clinical pathological features, such as tumor histological differentiation and lymph node metastasis. In OSCC cells, USP7 was shown to promote cell proliferation, inhibit apoptosis, enhance cell migration and invasion, and activate the Akt/ERK pathway. These effects all contributed to the development of OSCC. Further investigating the specific substrate protein that are modified by USP7 could provide a new direction for exploring the progression of OSCC.
